# Adolescent girls and young women’s (AGYW) access to and use of contraception services in Cape Town: perspectives from AGYW and health care providers

**DOI:** 10.1186/s12913-024-11236-0

**Published:** 2024-07-09

**Authors:** Tsidiso Tolla, Kate Bergh, Zoe Duby, Nandipha Gana, Catherine Mathews, Kim Jonas

**Affiliations:** 1 Health Systems Research Unit, South African Medical Research Council, Cape Town, South Africa; 2 School of Public Health, Division of Social and Behavioural Sciences, University of Cape Town, Cape Town, South Africa; 3 Department of Psychiatry and Mental Health, Division of Child & Adolescent Psychiatry, Adolescent Health Research Unit, University of Cape Town, Cape Town, South Africa

**Keywords:** Contraception services, Adolescent girls and young women, Health care providers, Sexual and reproductive health, South Africa

## Abstract

**Background:**

Access and use of contraception services by adolescent girls and young women (AGYW) remains suboptimal, exposing AGYW to early and often unexpected pregnancy. Unexpected pregnancies are a public health concern, associated with poor neonatal and maternal health outcomes, as well as school dropout, which may result in economic hardships. This study aimed to explore (a) AGYW perceptions and experiences of receiving contraception services from health care providers and (b) health care providers’ perceptions and experiences of providing contraception services to AGYW.

**Methods:**

Data were collected through semi-structured individual interviews with AGYW aged 15–24 years old and health care providers working in eight health care facilities around the Cape Town metropolitan area, in South Africa’s Western Cape Province. Thematic analysis was used to analyse the data.

**Results:**

AGYW and health care providers voiced varying, and often contrasting, perceptions of some of the barriers that hinder AGYW’s access to contraception services. AGYW indicated that provider-imposed rules about when to access contraceptive services hindered access, while health care providers felt that these rules were necessary for coordinating their work. In addition, AGYW highlighted health care providers’ hostile attitudes towards them as an important factor discouraging access. On the contrary, health care providers did not think that their attitudes hampered AGYW’s access to and use of contraception services, instead they emphasised that challenges at the health system level were a major issue, which they feel they have little control over. Such challenges made health care providers’ work unpleasant and frustrating, impacting on their work approach and how they receive and offer services to AGYW.

**Conclusion:**

The expectation of negative attitudes from health care providers continues to be at the centre of AGYW discouragement towards accessing contraception services. System challenges are among some of the key drivers of health care provider’s hostile attitudes, posing challenges to the efficient provision of services. In order to improve AGYW’s access to and use of contraception services, and subsequently achieve the country’s SDGs, conscious efforts need to be directed towards improving the workload and working conditions of health care providers.

**Supplementary Information:**

The online version contains supplementary material available at 10.1186/s12913-024-11236-0.

## Background

In Sub-Saharan Africa, adolescent girls and young women (AGYW) bear a disproportionate burden of unwanted and unintended pregnancy rates. Recent multi-level analyses by Ayalew [1] and Asmamaw [2] found that in Sub-Saharan Africa, the prevalence of unwanted and unintended pregnancies among AGYW was at 30% and 24.88%, respectively. Other evidence from the region also show that unexpected pregnancies remain persistently high among AGYW [3, 4]. Access to contraception can prevent unexpected pregnancies among AGYW, yet research in the Southern African Development Community (SADC) and Sub-Saharan regions shows that this population has poor access to contraception and in instances where contraception services are free, access does not match the contraception needs of AGYW [5, 6].

The International Conference on Population Development (ICPD) held in Cairo in 1994 enhanced the development of policies on the sexual and reproductive health (SRH) of AGYW. Following the ICDP, numerous regulations and guidelines on AGYW SRH have been developed both nationally and internationally with the hope of improving AGYW access to contraception services in order to prevent unexpected pregnancies and improve their overall SRH outcomes [7]. Currently, South Africa, like many countries is guided by the sustainable development goals (SDG), which aim to advance the health and well-being of all, setting out clear goals to advance the SRH and well-being of everyone [8]. SDG target 3.7.1 on contraceptive use seeks to ensure universal access to family planning by 2030. Global commitments such as the SDGs have increased AGYW knowledge on how to prevent pregnancy and in many instances, information on where and how to access contraception services in order to prevent rates of unexpected pregnancies, yet, such knowledge has not translated to increased use of contraceptive services [9–11].

Globally, AGYW’s access to contraception services is hindered by a complex array of multilevel factors. At the community level, AGYW’s perceptions of health care providers’ attitudes towards AGYW seeking contraception services have continuously been flagged as a major barrier to access. A recent study conducted in South Africa found that AGYW perceive health care providers to be ‘rude’ and ‘mean’, thus discouraging their access and use of contraceptive services [12]. In South Africa, several initiatives have been implemented to improve the manner in which contraception services are provided for AGYW. One example of these initiatives is the adolescent and youth friendly health services (AYFS) implemented by the Department of Health. In AYFS, the attitudes of health care providers are deemed key in the successful provision of SRH services to adolescents and young people. As a result, some studies in South Africa report shifts in health care providers’ perceptions of providing contraception services to AGYW [13]. Despite these shifts and all the initiatives – some spearhead by NGOs - that have been implemented to improve the provision of contraception and other SRH services, AGYW still believe they may not be treated well at health facilities, such a belief, which might be fuelled by rumours and the negative experiences of a minority, remains a barrier [14].

Evidence from across sub-Saharan Africa and further afield continues to document health care providers’ negative attitudes as one of the major barriers to AGYW access to SRH services [15–18]. Reasons for health care providers’ negative attitudes vary and are influenced by a number of different factors. For example, health care providers perceive AGYW to be young and irresponsible, creating feelings of hostility and resentment towards AGYW, and the desire to impose restrictions when providing contraceptive services to AGYW [19]. Additionally, health care providers fear that AGYW access to and use of contraceptives condones and encourages promiscuity and sexual activity [20–22]. Evidence suggests that health care providers employ tactics to actively discourage and dissuade AGYW from using contraceptives, such as being intentionally rude and unwelcoming to AGYW, and purposefully emphasising the side effects of contraceptives [23].

This study explores the relations between health care providers and AGYW during contraceptive service provision. Specifically, the study explores how AGYW and health care providers perceive one another during consultation for contraception services. Interpersonal dynamics and interactions between AGYW and health care providers are key in facilitating or discouraging contraception access and use. Understanding how AGYW experience and perceive health care providers and how health care providers in turn experience and perceive contraception service provision to AGYW can help us to identify key points of tension and thus serve as an entry point for policy makers and program developers seeking to improve AGYW access to and use of contraception services.

Several scholars warn of the problematic nature of the use of the terms unintended and unwanted pregnancies [24, 25]. Duby [24] reflects on the use of terms describing the discovery of unexpected pregnancy among AGYW, stating that the terms such as unintended and unwanted pregnancy are often used without consideration and investigation of how AGYW themselves describe or experience pregnancy intention and discovery. In acknowledgement of this, we have chosen to use the term ‘unexpected pregnancy’ in this paper.

## Methods

### Study design and setting

This qualitative study was conducted in low socio-economic communities in Cape Town, in the Western Cape province of South Africa. Participants for the study were recruited from eight public health care facilities purposively selected to include facilities that offered contraception, obstetric and abortion services. Selected health care facilities included public governmental facilities that provide contraception, obstetric and abortion care services. All facilities serviced relatively large, low socio-economic communities with relatively easy access for AGYW.

### Study population

Two study populations took part in this study: (1) AGYW aged 15–24 years, and (2) health care providers working in contraception and abortion care services. AGYW were stratified into two categories, (a) 15–17 years old and (b) 18–24 years old. Within these age groups, AGYW were further stratified into three sub-groups (a) AGYW who accessed contraceptives and did not have an unexpected pregnancy, to explore facilitators of contraceptive access and effective use; (b) AGYW who had an unexpected pregnancy, to explore experiences of AGYW who did not access or effectively use contraceptives or abortion services and to explore the barriers to access and effective use; and (c) AGYW who accessed abortion care services, to explore facilitators for access to abortion care services.

### Sampling

Data collectors approached AGYW at the health facilities. In doing so, they explained the study to AGYW and captured information of those interested using the recruitment spreadsheet. In addition to direct recruitment at the selected health facilities, health care providers who offer contraception services to AGYW were approached to help researchers recruit AGYW by asking their patients to give permission to be contacted for the purposes of the study. Health care providers who agreed were then given a study information sheet and recruitment spreadsheet to collect the names, contact details and preferred times to be contacted of AGYW who expressed an interest in participating. AGYW who were interested in taking part in the study were screened telephonically for eligibility, and if eligible, they were then enrolled over the phone, by trained data collectors who were proficient in participants’ preferred languages. Due to limited time and workload of health care providers, only four of the nine targeted health care providers were available and agreed to participate. A total of 67 interviews were conducted: 63 with AGYW, and four with health care providers.

### Data collection and procedures

Telephonic interviews, which lasted 30–60 min were conducted by experienced and trained female interviewers in participants’ preferred language, following an open-ended interview guide tailored for each specific sample group. Interview guides were developed for this study based on the theory of Patient Journey’s. The patient journey offered the opportunity to gather stories that matter to AGYW as users of contraception services and to link their stories to health service and health system issues [26, 27]. Three interview guides were developed for AGYW who had no unintended pregnancies (Additional file S1); AGYW who had unintended pregnancies (Additional file S2); and health care providers (Additional file S3).

Interviewers had post-high school qualifications and were trained on how to conduct interviews (over the phone) by an expert in qualitative research (ZD), the lead researcher (KJ) and the study coordinator (NG). Interviews were conducted telephonically due to COVID-19 regulations at the time, which included restriction of movement and small gatherings. Throughout data collection, interview questions and transcripts were continuously reviewed, and where necessary, modifications and adaptations were made. All interviews were audio recorded, with participants’ consent, and the interviewers took hand-written notes during the interviews. A questionnaire was also telephonically administered to record participants’ socio-demographic characteristics. Two questionnaires were developed, one for AGYW (Additional file S4) and the other for health care providers (Additional file S5).

### Data analysis

Audio recordings of interviews were transcribed verbatim. Interviews conducted in isiXhosa or Afrikaans were translated into English by an independent trained translator and quality checked for accuracy by the interviewers. Transcripts were uploaded into a computer assisted qualitative analysis software to assist with data organisation and verified by the third author (NG) who simultaneously compared each transcript to the audio. This process also allowed us to assess and identify when we had reached data saturation. Analysis involved a collaborative interpretation in which researchers (TT and KJ) immersed and familiarised themselves with the data through repeated readings in an active way searching for meanings and patterns. Transcripts were initially coded, using NVivo by TT, followed by discussion by TT and KJ, after which codes were modified where necessary.

### Ethical considerations

Ethical approval for this study was granted by the SAMRC Research Ethics Committee (REC), REC ref: EC044-10/2021. The Western Cape Department of Health granted permission to interview health care providers. Permission to access health facilities was sought from and granted by facility managers. All eligible study participants were informed about the purpose of the study in their preferred language prior to enrolment. Participation in the study was voluntary. Prior to conducting any interviews, parents or caregivers of AGYW younger than 18 years of age granted consent telephonically. Following this, verbal informed consent was obtained from each AGYW telephonically. Permission was also sought from all participants to audio record the interviews. Participants were reimbursed with R150 (7.96 USD) for their time.

A plan for managing distressed participants was put in place for participants who showed signs of discomfort and stress. Participants were reminded that they did not have to answer questions that made them uncomfortable. Measures to report disclosure of sexual abuse and deliberate neglect of a minor to community services equipped to manage situations of child abuse and sexual abuse, such as social workers were put in place. To maintain confidentiality, all information was uniquely identified and anonymised, recordings of consent and interviews were safely stored in the SAMRC password-protected project drive.

## Findings

A total of 67 participants took part in this study, 63 AGYW and four health care providers. Table 1 presents characteristics of AGYW who took part in this study. More than two thirds of the AGYW where in the 18–24 years bracket (*n* = 45). Most AGYW identified as heterosexual (*n* = 61). Xhosa was the predominant language spoken by participants (*n* = 48). In terms of education, the majority had a secondary education (*n* = 50). There were more AGYW who had never been pregnant (*n* = 44) compared to those who had been pregnant (*n* = 19). Among those who had been pregnant three AGYW had more than one pregnancy, thirteen had been pregnant once and three preferred not to respond to the question on how many pregnancies they had had. Further among AGYW who had been pregnant, six had had an abortion.

**Table 1:** AGYW demographics

Variables	Participants (*N* = 63)		
**Age (15–24 years)**			
15–17 years	18		
18–24 years	45		
Gender	All Female		
**Sexual orientation**			
Bisexual	2		
Heterosexual	61		
**Language**			
IsiXhosa	48		
Afrikaans	8		
English	5		
Sesotho	1		
IsiNdebele	1		
**Highest level of education**			
Primary school	3		
Secondary school	50		
University/TVET college/other tertiary institution	10		
**# of AGYW who had been pregnant**			
Yes	19	***number of pregnancies***	
		More than one	3
		One pregnancy	13
		Prefer not to answer	3
No	44		
**# of AGYW who had been pregnant and gave birth**			
Yes	13		
No	6		

A total of four health care providers (aged 20–50 + years) took part in this study. All health care providers were female, two had a postgraduate degree, one a diploma and one a higher certificate. Health care providers spoke IsiXhosa (*n* = 2) and Afrikaans (*n* = 2). Three health care providers were employed full-time and one was employed part-time. Three were married and one was single. Of those who were married, two had children.

The study was conducted during the COVID-19 pandemic and the key findings of the study reveal that during the time the study was conducted, AGYW could not access contraception services whenever they wanted to as access was limited to two days per week. Even when AGYW did present to the health facility on the date and time reserved for them, they had to wait for long periods of time to be attended to by a health care provider. AGYW described negative experiences with health care providers, largely characterised by hostile attitudes. Some health care providers were cognisant of the challenges faced by AGYW at the hands of their colleagues, as such, their focus was protecting the rights of AGYW and improving their experiences when accessing contraception services. Below, three key emergent themes are presented: (a) Provider imposed barriers towards AGYW’s access to contraception services (b) Long waiting times, and (c) Health care provider’ hostile attitudes.

### Provider imposed barriers towards AGYW’s access to contraception services

Findings showed that each health facility had its own set of rules and regulations in order to effectively and efficiently provide health services to the community. As a result, AGYW were restricted to a specified date and time in which they were able to access contraceptive services. AGYW described experiences where they were turned away simply because they had decided to access contraception services in the afternoon while not wearing their school uniform. Although the “rule” was that AGYW could access services in the afternoon on their specified dates, they could only do so if they were wearing school uniform. From the sample of AGYW who had accessed contraceptive services and did not get pregnant, one participant stated that: “It doesn’t make it easy…to be assisted… because when you are not wearing school uniform and you get there at 2pm or 3pm. They will ask where you come from then you say you are from school, then they will ask why you came not wearing school uniform, “how will we know if you’re coming from school? Where were you all this time?” They will ask questions like that, and say they can’t help you, you must come the following day around 8am”.

Health care providers confirmed that indeed health facilities had set rules about when to assist AGYW, as illustrated in the quotes below.

“[…] for those that go to school, we usually prioritise them, that after school they can come because we dedicate time for them from 2 o’clock up until 4 o’clock. They can come wearing (school) uniform” (Health care provider).

“[…] because the protocol says they come on Tuesday and Thursdays after school in their (school) uniforms” (Health care provider).

“[…] When schools are opened which means they must not go to school but to the clinic for family planning. They must go to school then after school go to the clinic, they know that family planning is on Tuesdays for them, it is also on Thursday afternoons. When schools are closed, they know they don’t come in the afternoon because obviously schools are closed” (Health care provider).

While from the perspective of the clinic management it might make sense for facilities to have specified times for when they can provide AGYW with access to contraception services, the rigidity of these protocols makes it challenging for AGYW who are unable to access the clinic at the set time or date, and whilst wearing school uniform. In the following quotation, one AGYW who accessed contraceptive services and did not get pregnant recounts how she was not assisted simply because she was not wearing school uniform, despite having a valid reason:

“They (health care providers) like to speak out of turn, the last time I went (to the clinic), I left my card at home, I came back and took off my (school) uniform. I was not assisted on that day because that lady said I know that I am not supposed to take off my (school) uniform, and I know but I went there early it was around 2pm. She just took the card and wrote another date and threw the card back at me”.

Health care providers were not unaware of how limiting and restrictive the protocols can be, as such, some health care providers said that they would assist AGYW who did not come at the specified time, as illustrated in the quote below:

“Some things are not in my power, and I don’t know how to deal with other things. But I don’t want to lie, when they get a chance they come even if it’s not a Tuesday because on that day they had afternoon class, “I was supposed to come on Tuesday but I had an afternoon class and now its Friday we finished early” I usually give the family planning and not even bother about the folder because I know that she will struggle to get the folder. I would inject her, to me what is important is getting what they want” (Health care provider).

### Long waiting times: a systemic challenge or health care provider-imposed challenge?

Having to spend longer periods of time at the health facility was a challenge cited by many AGYW who reported that a key barrier to accessing contraception services was fear of long waiting times. AGYW perceived long waiting times to be caused by health care providers’ laziness. In the following quote, one AGYW participant who had been pregnant recounts how she visited a health facility for contraceptives and was made to wait for a long time even though there were health care providers who were “free”: “There were nurses but they were not doing anything, it is free around 3pm and you find them sitting but we will wait for more than an hour at the clinic, so I ended up not going”.

On the issue of long waiting hours, health care providers were aware that AGYW did not like to spend prolonged periods of time at the health facility. However, they said that sometimes, procedures made it hard for them to assist AGYW as quickly as possible. In the quote below, a health care provider narrates how the process they need to follow before attending to an AGYW prolongs the time AGYW may spend at the clinic:

“Another challenge that we have is that [health department] is all about Stats (statistics), it’s all about numbers. You get one nurse on the family planning, understand. And I don’t wait for school kids, I do adults so long. Now, all that process starts from pulling out of folders because obviously everything must be captured down on the folder, it’s all about numbers, they don’t really care how it’s done but it must be done… that also take time” (Health care provider).

Health care providers shared multiple frustrations that they have to deal with in their day-to-day work. One of these was the emphasis on quantity over quality, which was regarded as particularly frustrating considering that there was a shortage of staff, as described in the excerpt below:

“[…] one cannot assume you can just walk in and be assisted, due to insufficient staff being available to assist. It can be very difficult at times, but we try our best to accommodate as many as we can. When they (AGYW) arrive, they are immediately directed to the counsellor” (Health care provider).

Although some health care providers tried to do their work despite the shortage of staff, some seemed to shift the blame to AGYW for coming close to the closing time or when they are in meetings, thus resulting in AGYW waiting a long time to be assisted, as illustrated in the quote below:

“You will find most of them (AGYW), they come when it’s close to 4 o’clock. And then sometimes they find us in meetings or maybe there is something else that we are busy with, then we don’t have time to attend to them at the time. They are therefore not patient and that process is tiring at the clinic” (Health care provider).

In order to mitigate system challenges, health care providers suggested that the Department of Health should work in collaboration with the Department of Education to provide services in schools, which they believed could potentially decrease the rates of unexpected pregnancies among AGYW:

“If it was about me, you know previously we used to have, relationships with schools that are closer to us, understand? Certain days a week a nurse will go to schools to make sure that girls get family planning” (Health care provider).

“I come from Pretoria, and we provided family planning services at three of the four high schools. We would go after school, at about 3’oclock. We saw them one by one in a small venue on the sports field. Young girls would come to us, and we provided them with the various family planning methods, and it worked very well” (Health care provider).

In addition to long waiting times, AGYW participants complained that setting up an appointment did not help mitigate the issue of spending prolonged periods of time at the health facility. Even when AGYW set appointments, they explained that they still had to wait for long periods before being attended to. In the quote below, an AGYW respondent who had accessed contraceptive services and did not get pregnant, explains her frustration at not being attended to at the time her appointment was set:

“The challenges that I encountered- there’s only one (health care provider), when you do arrive at the clinic… you wait a bit. Even though maybe… your appointment is at 11:30, you will wait today even though it is 11:30. I do expect when the time is set to 11:30, I expect to be given service at exactly 11:30. I do not expect to wait because I was not late nor early for my appointment. Sometimes maybe if … I have an errand to run after the clinic. It gets irritable, because you’ve been waiting, and you still have to do something else. So, uhm it is a bit challenging there”.

The AGYW in the extract above seems aware of her rights as a service user that she should not have to wait for too long, particularly when one has an appointment scheduled. However, system-level challenges such as understaffing and over-crowded clinics make it hard to create a welcoming environment and for health care providers to provide sufficient counselling and care to AGYW presenting for contraception services. In the excerpt below, the health care provider articulates how she wanted to do more than just give contraception services, wishing that she could build rapport and connect with the AGYW, but due to system challenges, this became a challenge:

“I do not think it is youth friendly. These days things are hectic at the clinic. It is as if every day just gets busier than the previous day. The current staff cannot cope with the number of patients that has to be attended to. So, the time that you are going to spend with someone, especially when it is a young person, you really need to sit down take your time and talk and show you are interested. I think when one shows interest then that young person will return every time, but there isn’t time to sit, you need to build up a relationship with them, but there isn’t time to really do that because within ten minutes everything must be handled or else, we will not get to accommodate everyone, and I think that in itself creates a very cold atmosphere” (Health care provider).

### Health care provider’s hostile attitudes

In addition to system challenges, such as limiting when AGYW could access contraception services at the facilities, AGYW experiences were further impacted by health care providers’ hostile and negative attitudes. AGYW respondents who had accessed contraceptive services and did not get pregnant views health care providers as rude, which increased AGYW reluctance to access contraception services.

Every time when one goes there (clinic), they shout but then sometimes it’s okay because when you get there, you get tested for HIV and pregnancy and then when they inject you it’s painful and it gets sore for days. And sometimes it’s very difficult to go to the clinic because nurses are rude.

In some instances, AGYW mirrored health care providers’ negative attitudes. For example, some AGYW said they did not submissively accept health care providers’ rude attitudes, but instead would want to respond in a defensive and rude manner because they do not like being treated in that way. One AGYW respondent who has accessed contraceptive services and did not get pregnant stated that: “Most of us have respect, so when one goes there and get a rude nurse you lose respect. I wish nurses can speak nicely with us because we get tired and we think if we respond to their rudeness, they will not assist us. I wish they can speak nice to us …that is why most girls don’t like to go there (clinic) because nurses are rude and they would ask questions and you answer. If it is not an answer that she expected or something then she will have an attitude and inject you not so nicely.”

Such tension can create a potentially toxic environment for AGYW to receive contraceptive services and for health care providers to deliver these services. This may also result in health care providers failing to offer proper counselling during a consultation as they may feel attacked or even disrespected. Health care providers were aware of the fact that some of their colleagues were rude towards AGYW presenting at the health facilities for contraception services. It also seems health care providers were aware that AGYW tended to mirror their attitudes, as narrated by the health care provider in the following quote:

“okay, (deep sigh), it’s nice to work with them (AGYW), they come to the level that you approach them with, okay I am a very bubbly person. I’m a very… what can I say? My mood is always good. If you talk about them to me, they will tell you that friend, they call me friend and I call them friend” (Health care provider).

There were instances when health care providers infringed AGYW’s right to information. AGYW felt that they were not given sufficient information about the different kinds of contraceptives and how they work for them to make informed decisions about the choice of contraception they preferred, as narrated in the quote below from an AGYW respondent who had accessed contraceptive services and did not get pregnant: “It (contraceptive method) was chosen for me because they (nurses) never asked me, they just injected me and then when I checked the card, I saw it was Nuri (Norethindrone oral contraceptive pill)”.

Health care providers were aware that some of their colleagues did not provide AGYW with sufficient information in order to make informed choices regarding which contraceptive method to use. In the quote below, a health care provider talks about how some of her colleagues tend to give all young girls the injection without asking them about their preferred method.

“Some of the nursing sisters say that if you are 14 or 15 years old, you immediately get given the injection. Which I feel is not right, because you have a choice.” (Health care provider).

There were serious repercussions for health care providers’ failure to inform and educate AGYW about the different types of contraceptive options, how they work and the waiting period before one can be protected from pregnancy. In the following quotation, an AGYW respondent who had been pregnant explains that because she was not aware that there is a waiting period for the method to be effective, she discovered that she was pregnant after accessing contraception services: “so that is how I got pregnant. I went to the clinic again in the following month for family planning while I was already pregnant but I did not know…I got contraceptives then I found out later that no I am pregnant and since I was not getting my periods it was not easy to know as I was on contraceptives”.

In addition, health care providers were aware of the potential consequences of their negative attitudes towards AGYW access to contraception services. In the quote below, the health care provider talks about how AGYW who receive positive treatment honour their next appointments.

“It depends on the facilities and also with the person who is working with them (AGYW). Because if you are friendly with them, you communicate, they don’t mind coming to the clinic. So, it also depends with the individual who is dealing with them.” (Health care provider).

When AGYW experience a positive and welcoming environment and positive attitudes from health care providers, they are likely to be more motivated to continue attending the clinic. Health care providers expressed an awareness of this, as such, they said that notwithstanding their frustrations as a result of system challenges, it was important to try and always remain calm and be cognisant of how the manner in which they speak to AGYW can determine whether they return to the health facility or not:

“At the beginning of the month, I was a little stressed. Yesterday, I saw a patient I had seen a while back and she told me she wondered if I was better now because the last time she was at the clinic I was so tough and she doesn’t know me like that. I didn’t even realise at the time, that I was like that, so one needs to be so careful. One should just remind yourself to be calm, especially when you already seeing patient number 52. …especially with young people. You must be aware of what you say and how you say it. I think it is not so much what you say, but how we say it that will determine if that person returns to the clinic” (Health care provider).

## Discussion

This study explored the interpersonal relationship dynamics and interactions between AGYW and health care providers during contraception service provision at health facilities in low socio-economic communities in Cape Town. Specifically, the study explored how AGYW and health care providers perceive and view each other when AGYW attend health facilities to access contraception services. The findings show that there remain significant challenges in AGYW access to contraceptive services. AGYW highlighted systematic challenges such as long waiting times, provider-imposed rules that AGYW could only access contraception services on certain days and specific times and health care providers’ hostile and negative attitudes. Health care providers identified system-level challenges that made their work unpleasant and led to frustrations, which seemed to impact how they receive and offer services to AGYW and their overall work approach.

A closer analysis of the challenges identified by AGYW and health care providers shows that system-level operations and priorities are key in shaping relations between health care providers and AGYW during contraceptive service provision. The manner in which health care providers approach the provision of contraception services to AGYW has serious implications for AGYW experiences when they present for these services. It appears that the point of contention between AGYW and health care providers when AGYW present to health facilities for contraception services relates predominantly to system level challenges. Given this, the findings of the current study challenge the assertion that health care providers’ attitudes towards AGYW who present for contraception services are simply due to cultural and religious beliefs and lack of training. The findings highlight that in South Africa, the manner in which the public sector health system is set up has serious implications for both health care providers and AGYW.

Previous research has described the negative effects of system challenges on health care providers’ approach towards AGYW presenting for contraception services. Jonas [28] found that health care providers did not set out to be rude to AGYW, however, frustrations from systems challenges negatively impact their attitudes. Similar sentiments were shared by health care providers in the current study, who said that the Department of Health’s targets are driven to attend to a high volume of patients, thus compromising the quality of care. When health care providers feel hurried and under pressure they may be unable to provide sensitive care [29]. In a recent study, Hancock [30] emphasises the need to explore and understand the full breadth of factors, including contextual and systematic factors that may influence the behaviour of health care providers. The authors further suggest the need to move away from blaming health care providers to actively supporting them in doing their jobs and managing their workloads [30].

In order to manage their workload, deal with the overwhelming number of patients in a day and find ways to dedicate attention towards AGYW, health care providers in the current study enforced the rule that AGYW could only access contraception services on Tuesday and Thursday, afterschool, before 16:00. In low and middle income countries, it is not uncommon for health services to be “organized around the functional separation of services, either by day of the week (for example, ‘HIV treatment initiation day’ or ‘family planning day’)…” [31]. The manner in which health care providers do this in order to cope with their day-to-day work and organise their workload is also reported in previous research. For example, research on TB care in South Africa report on how health care providers may adopt certain rituals and strategies as they care for TB patients and may need to find ways to organise their workload in order to make their work manageable [32, 33]. Within this management, it is important to note that the manner in which workers manage their workload might inadvertently lead to a failure to consider the needs of patients as has been observed in other studies [31, 32].

Findings from the current study show that in the process of health care providers wrestling to manage their daily workloads whilst meeting Departmental targets, they inadvertently discouraged AGYW from accessing contraception services. Some AGYW shared that health care providers refused to provide contraception services to them under the guise of clinic protocols. Some shared that health care providers did not like it if they visited the health facility at 15:45, which is still within the stipulated time frame according to the protocol. Although protocols on how and when AGYW can access contraceptives may not be out of malice or as a result of negative intentions from health care providers [34], they are still restrictive and limit access and may be perceived by AGYW as a consequence of a negative attitude among health care providers towards them. Such protocols or what Calhoun [35] has termed provider-imposed restrictions, have the potential to set back efforts and progress on AGYW access to contraceptive services.

The rule that AGYW can only access contraception services twice a week before 16:00 whilst wearing school uniform is restrictive and may result in AGYW dropping out of family planning if they miss their designated dates. This is problematic because AGYW already face a multitude of challenges when accessing contraception services [16, 18, 19]. To be refused services simply because there is a protocol in place that stipulates that AGYW can only be assisted at certain times when dressed in a certain way is an unnecessary stumbling block. Also, considering that by the South African law, AGYW are allowed to seek contraception services, refusal to assist AGYW who present for contraception services can be classified as unethical behaviour as this may dissuades these young girls from protecting themselves from the harms of pregnancy [12].

Previous attempts to increase AGYW access to and use of SRH highlight the need for youth-friendly health services (YFHS) and training of health care providers, yet data on the effectiveness of YFHS and the training of health care providers is inconclusive. Studies in Africa show that YFHS may have a positive impact on SRH access but is not cost-effective and faces several challenges on implementation [36, 37]. Similarly, studies show that training health care providers to equip them with the necessary skills to provide AGYW with contraception services may have a positive impact in the manner in which they provide SRH services to AGYW [38–40]. However, even in settings where training of health care providers may promise positive outcomes, such interventions may not work if health care providers’ hostility is a by-product of challenges within the health system [41].

### Limitations

The study has a number of limitations. First, the findings of this study should not be generalised to all AGYW and health care providers. Second, the number of health care providers interviewed for this study was small (*n* = 4). Third, health care providers may have been more likely to express supportive attitudes to AGYW. Fourth, some AGYW were identified through health care providers, and this could have resulted in response bias. Despite these limitations, these findings provide useful information about the interactions between AGYW and health care providers during contraception service provision and can help to identify points of tension and to guide future intervention efforts.

### Implications

The findings of this study have some important implications for research and policy. First, priorities at the Department level need to clearly recognise AGYW’s access to and use of contraception services as a key mandate. The department might need to have clear deliverables around AGYW’s access to contraception services, this may signal to health care providers that this population is a priority and may thus energise health care providers in their approach when interacting with AGYW. Also, as health care providers seek to find ways to manage their daily workload, having a clear mandate regarding AGYW’s access to contraception may drive health care providers to find ways that increase AGYW’s access to contraception.

Second, the Department of Health may need to consider youth friendly nurses - stationed in standard of care health facilities - who are responsible for all youth related SRH issues, including contraception services. Having a youth friendly nurse might over-time allow youth to build a closer relationship with the youth friendly nurse potentially leading to open discussions around young people’s SRH concerns. Also, having youth friendly nurses per health facility might mean that these nurses would not out rightly mistreat and hide behind the “all nurses are rude” narrative as they would easily be identified. Further, there may be a need to train health care providers on the delivery of comprehensive SRH information and services, empowering AGYW in SRH decision-making.

Third, in order to improve AGYW’s access to and use of contraception services, and subsequently achieve the country’s SDGs, conscious efforts need to be directed towards improving the workload and working conditions of health care providers. We acknowledge that the issue of high workload and poor working conditions is a problem faced by the health care system of South Africa and that the department of health in South Africa faces significant challenges with the high burden of care imposed by the long-term needs of the over eight million people living with HIV in the country. Also, provincial and national department of health need to have a clear mandate on AGYW’s access to and use of contraception services. As health care providers wrestle to manage their workload with poor resources and a large number of patients to attend to, being courteous and welcoming might not be prioritised as highly as than services to a certain number of patients in order to meet their goals as a health facility.

## Conclusions

The findings from this study indicate that health care provider and health system factors remain major constraints on South African AGYW’s access to and use of contraception services. AGYW use of contraception services is dependent on the attitudes and behaviours of health care providers, which are dependent on their working conditions, workload and priorities as mandated by the Department of Health. AGYW’s access to contraception services is a global priority and critical in reducing rates of early and unintended pregnancies and in turn improving AGYW’s educational and economic opportunities and overall wellbeing. However, the realisation of this dream may be hampered or delayed if AGYW continue to perceive or experience mistreatment from health care providers during provision of contraception services. AGYW’s use of contraception services is dependent on the behaviour of health care providers, which in itself is dependent on attitudes and skills of health care providers, as well as improvements in their working conditions. Focusing interventions on these factors may help to bring South Africa closer to achieving the SDGs.

### Electronic supplementary material

Below is the link to the electronic supplementary material.

Supplementary Material 1

Supplementary Material 2

Supplementary Material 3

Supplementary Material 4

Supplementary Material 5

## Data Availability

The data cannot be shared openly, to protect study participant privacy. However, the data can be made available from the authors upon reasonable request.

## Abbreviations

ICPD: International Conference on Population and Development
SRH: Sexual and Reproductive Health
SDG: Sustainable Development Goals
AYFHS: Adolescent and Youth Friendly Health Services
REC: Research Ethics Committee
SAMRC: South African Medical Research Council
