# Supplementary material for: Adolescent girls and young women’s (AGYW) access to and use of contraception services in Cape Town: perspectives from AGYW and health care providers
Source: BMC Health Serv Res. 2024 Jul 9;24:787. doi: 10.1186/s12913-024-11236-0 (PMC11234529; doi:10.1186/s12913-024-11236-0)
Supplement: Supplementary file 1 — Supplementary Material 1 [file 12913_2024_11236_MOESM1_ESM.docx]

**Interview guide with AGYW** **who accessed contraceptives and did not have an unintended pregnancy**

**INTERVIEWER READS:**

Thank you for agreeing to be part of this study and to take part in this interview. Our goal is to hear the stories and experiences of adolescent girls and young women like you who have accessed and used contraceptives from the health services. We are interested in hearing about your journey accessing contraceptives. There are no right or wrong answers we would like to hear your story. To begin:

1. We are interested in hearing about your journey from the time you started thinking about family planning through the process of navigating the clinic when you went to get contraceptives.
   - Please tell us your story, how it came about for you to think you might need family planning.
     - Probe for persons spoken to about family planning. Boyfriend? Family/friends? Others like teacher…?
   - What made you decide to get family planning?
     - What things/people encouraged you to get family planning?
     - What things discouraged you from getting family planning?
     - Probe on religion, culture, community, peer, family influences
2. We found out that AGYW choose to use family planning for different reasons. In your journey, did you (do you) personally feel you need to use family planning?
   - Probe: can you please tell us why did you (do you) feel you need to use family planning? Or why did you (do you) not feel you need to use it?
3. How would you describe your journey getting to the clinic to get family planning?
   - What made it possible for you to come to this clinic?
   - What made it challenging to come to the clinic?
4. How would you describe the clinic atmosphere when you got there?
   - How would you describe the people you talked to at the clinic to help you get family planning? How did the receptionist/nurse treat you?
   - How would you describe your experience at the clinic where you went to get family planning? How does it compare to what you had expected?
     - Probe for challenges and facilitators
5. Please tell us more about the specific method of contraception you are using.
   - How did you come to use this method?
   - Did you already know about this method before you used it? Please tell what you know about this method and your understanding of how it works.
   - Where did you get the information about this method?
6. Can you tell us about other methods of contraception that you are aware of?
   - How did you come to be aware of these methods?
7. Have you talked to your parents or caregiver about family planning?
   - Can you share your experience when you started talking to your parents about contraceptives?
   - Have you talked to other women like your sisters, cousins, or neighbors? Can you share your experience talking to them? How does this experience compare when talking to your parents/caregivers?
8. Are you currently in any relationships?
   - Have you spoken about contraceptives with your partner/s?
   - Please tell us how you and your partner/s started talking about family planning?
   - Was your partner involved in your decision to use family planning?
   - What role has your partner played in your decision to use family planning?
     - Probe for support or disproval
9. What would you say has made it easy for you continue using family planning?
   - Probe for facilitators of access and use
10. Overall, how you would say your experience to accessing and using family planning was?
    - Probe for positive or negative experiences
11. Please tell your story on how it was for you during COVID-19 and the lockdown when you needed contraceptives or other family planning services.
    - Probe for whether she wished to use, or used contraceptives during lockdown?
    - Probe for what made it difficult or easy to get contraceptives
12. Please share with us what do you think makes it difficult for other young women like you, to use contraceptives
    - What makes it hard for them to continue using them?
    - What do you think can be done to motivate them to continue using contraceptives?
13. Do you have any other comment or question you would like to ask?
