# Supplementary material for: Adolescent girls and young women’s (AGYW) access to and use of contraception services in Cape Town: perspectives from AGYW and health care providers
Source: BMC Health Serv Res. 2024 Jul 9;24:787. doi: 10.1186/s12913-024-11236-0 (PMC11234529; doi:10.1186/s12913-024-11236-0)
Supplement: Supplementary file 2 — Supplementary Material 2 [file 12913_2024_11236_MOESM2_ESM.docx]

**Interview guide with AGYW who had an unintended pregnancy**

**INTERVIEWER READS:**

Thank you for agreeing to be part of this study and to take part in this interview. Our goal is to hear the stories and experiences of adolescent girls and young women like you, and how you have experienced family planning services and pregnancy. We are interested in hearing about your journey that brought you to where you are now, having given birth to a baby. We want to hear your story. To begin:

1. We are interested in hearing about your story on how you became pregnant.
   - Before falling pregnant were you using any family planning? Please tell us about that experience until you became pregnant. With a No response: Please tell us your story on how you became pregnant.
2. How did you find out that you were pregnant?
   - Please tell us the events before you fell pregnant.
   - Please tell us what you think happened that led to you falling pregnant
3. How did you feel discovering that you were pregnant?
   - Please tell us your experiences when finding out that you were pregnant
4. Who did you tell about your pregnancy and how did they respond when you told them?
   - Probe for reaction from parents/ school/ boyfriend/ peers
5. Before you became pregnant, what was your experience with the family planning services and methods? Were you using any family planning when you found out you were pregnant?
   - Probe for whether used, and why or why not using family planning
   - Probe for motivations around family planning?
   - Probe for challenges and facilitators in accessing family planning
   - Probe for challenges in adhering to use of family planning methods
   - Probe for experience with different methods and thoughts about the methods, how they work, and side effects.
6. Before you fell pregnant were you using any family planning methods?
   - Which family planning method were you using? How did you come to use this family planning method? Did you stop using this method? Why?
   - Please tell us what you know about this method and how it works in your understanding. Where did you get the information about this method?
   - Are you aware of other family planning methods? Please elaborate
   - Where did you hear about the other family planning methods? How did you become aware of these?
7. Did you ever think about terminating the pregnancy?
   - Probe for motivations around abortion?
   - Probe for whether you knew where to go for abortion care services if you wanted to terminate the pregnancy?
   - Where would you have gone to? How did you come to know about this place?
   - Probe for access challenges, and facilitators, e.g. health system, family/cultural/religious beliefs.
8. Would you say the pregnancy has changed your life in a way?
   - How has it changed your life?
   - Probe for schooling, for family planning access and use, family relationships, boyfriend and peers’ relationships
9. Please tell your story on how it was for your during COVID-19 and the lockdown when you wanted to make decisions about getting pregnant or preventing pregnancy.
   - Probe for barriers to accessing contraceptives and abortion services
10. Please share with us what do you think makes it difficult for young women like you to be in control of decisions about whether they get pregnant or not.

- What makes it hard for them to continue using them?
- What do you think can be done to motivate them to continue using contraceptives?

1. Do you have any other comment or question you would like to ask?
