# Supplementary material for: Adolescent girls and young women’s (AGYW) access to and use of contraception services in Cape Town: perspectives from AGYW and health care providers
Source: BMC Health Serv Res. 2024 Jul 9;24:787. doi: 10.1186/s12913-024-11236-0 (PMC11234529; doi:10.1186/s12913-024-11236-0)
Supplement: Supplementary file 3 — Supplementary Material 3 [file 12913_2024_11236_MOESM3_ESM.docx]

**Interview guide for Health service providers**

Position in the facility:______________________________

**INTERVIEWER READS:**

Thank you for agreeing to be part of this study and to take part in this interview. Our goal is to understand the family planning services provided to adolescent girls and young women aged 15-24 years in this facility. We are interested in hearing your experiences and views as a service provider on providing contraceptives and abortion care services to AGYW. To begin:

1. How would you describe the AGYW coming to your facility for contraceptives and or/ abortion care services?
2. What are your views on contraceptive use by AGYW?

- Adolescent girls aged 15 – 18?
- Young women aged 19-24?
- Do you think it’s a good idea for them to use contraceptives? Why or why not?
- What do you think they need in relation to contraceptives? Probe for information /knowledge provision of contraceptives…

1. How would you describe AGYW’s adherence to contraceptive methods?

- Why do you think that is?
- Probe for perceived challenges in contraceptive adherence from service providers’

1. What do you think can be done to prevent unintended pregnancies among AGYW?

- How can the clinic promote contraceptive use among AGYW?
- What do you think AGYW need to adhere to their contraceptive methods?
- How can AGYW be motivated to continue using contraceptives consistently?

1. Do you provide abortion care services in this facility?

- What are your views on providing abortion services to young women?
- Probe for personal, cultural/ religious views…

1. How would you describe your services for AGYW in this facility?

- To what extent would you say this facility provides youth-responsive and friendly services for AGYW? Please describe how this is achieved.
- To what extend would you say this facility prioritizes family planning services?
- What about family planning services for AGYW?
