# Supplementary material for: Adolescent girls and young women’s (AGYW) access to and use of contraception services in Cape Town: perspectives from AGYW and health care providers
Source: BMC Health Serv Res. 2024 Jul 9;24:787. doi: 10.1186/s12913-024-11236-0 (PMC11234529; doi:10.1186/s12913-024-11236-0)
Supplement: Supplementary file 4 — Supplementary Material 4 [file 12913_2024_11236_MOESM4_ESM.docx]

**Demographic information form AGYW**

Record ID:_____________________________

PTID:_____________________________

**INTERVIEWER READS:**

Thank you for being a part of our research. As you know, people live in different places, with different customs, cultures, sexual practices, and beliefs. We hope to include people from different communities in our research. We respect all people. Not all questions we ask in our research will apply to you. Because we do not want to make assumptions, we ask the same questions to everyone. We want you to be comfortable in speaking with us. You do not have to answer any question that makes you uncomfortable.

The following are some basic questions regarding your background to help us know what type of people participated in the discussion for this study. All the information you provide will be kept confidential and will not be shared with anyone else besides the research study staff.

Now I am going to ask you some questions about yourself. The answer to these questions will tell us more about who you are, such as your age and ethnicity. I will also ask you about your sex and gender. Please feel free to ask any questions about things that you don't understand.

1. What is your date of birth?__________________

(If DOB unknown please enter age below)

1. What is your age?__________________________

The next question is about your sex. When I ask about your sex, I am asking about what sex you were determined to be at birth, which is generally done by looking at a baby's genitals (sex organs).

1. What sex were you assigned at birth?

- Male
- Female
- Intersex
- Prefer not to answer

The next question asks about gender. Gender is the social part of being male or female, and relates to your self-identity. I am asking whether you consider yourself to be male, female, transgender male, transgender female, gender variant, or if you identify yourself in an additional category.

1. How do you identify your gender?

This item must be self-reported by the participant. Site staff are encouraged to document in chart notes if the participant, during study participation, prefers to be referred to by a specific pronoun or gender.

- Male
- Female
- Transgender
- Gender variant
- Prefer not to answer
- Other self-identity

Please specify:_________________________________

1. How do you identify your sexual identity?

- Straight/Heterosexual
- Gay/Homosexual
- Lesbian
- Bisexual
- Not sure/Undecided
- Prefer not to answer
- Other

Please specify:_______________________________

1. What is the language most spoken at home?

- IsiZulu
- Sesotho
- IsiXhosa
- Setswana
- Siswati
- English
- Afrikaans
- Other

Please specify:_______________________________

1. Who do you currently live with?

- Parent/s / mother / father
- Grandparent/s
- Main/steady spouse or spouse
- Casual partner
- Other family member/s
- Non-family / friend/s
- Alone
- Prefer not to answer

((Mark all that apply))

1. Name of area / location where you currently live: _________________________
2. Have you had any pregnancies?

- Yes
- No
- Prefer not to answer
  1. If yes, how many pregnancies have you had?_________________________

1. Have you given birth to any children?

- Yes
- No
- Prefer not to answer
  1. If yes, how many children have you given birth to?______________________

1. Are you currently taking care of any children?

- Yes
- No
- Prefer not to answer
  1. How many total children are you currently taking care of?________________

1. What is your highest level of education?

- No schooling
- Primary school, not complete
- Primary school, complete
- Secondary school, not complete
- Secondary school, complete
- Attended college or university
- Prefer not to answer
- Other

((mark one))

Please specify:________________________________

1. Do you currently earn an income of your own?

- Yes
- No
- Prefer not to answer

1. During the last 12 months, where did you get your income or financial support?

- Formal Employment (working for company, working in a shop, etc.)
- Informal Employment (recycling, piece work, selling goods/clothes, gardening, etc.)
- Self-Employment
- Social grants (child support, disability, etc.) Main/steady spouse or partner
- Casual partner
- Child Maintenance
- Family (other than spouse)
- Friends
- Selling sex for money
- Exchanging sex for goods (not money)
- Prefer not to answer
- None of the above
- Other

((mark all that apply))

Please specify:_________________________________
